# Effects of dairy manure biochar on adsorption of sulfate onto light sierozem and its mechanisms

**DOI:** 10.1039/c8ra08916g

**Published:** 2019-02-12

**Authors:** Baowei Zhao, Huan Xu, Fengfeng Ma, Tao Zhang, Xujun Nan

**Affiliations:** School of Environmental and Municipal Engineering, Lanzhou Jiaotong University No. 88, West Anning Road Lanzhou 730070 Gansu P. R. China zhbw2001@sina.com baowei.zhao@yahoo.com +86-931-4956017 +86-931-4938017

## Abstract

The adsorption of nitrogen and phosphorous nutrients on biochar and even biochar–soil mixtures was investigated. However, the situation of sulfur was not very clear. Here, sulfate (SO_4_^2−^) adsorption onto dairy manure biochar obtained at 700 °C (DMBC700), soil (light sierozem) and a 1 : 9 (w/w) biochar–soil mixture (DMBC700-soil) was investigated using batch experiments. The contact time, sulfate concentration, and solution pH value were chosen as the main factors; their effects on sulfate adsorption were tested, and the kinetics and isotherms were also investigated. Fourier transform infrared (FTIR) and X-ray diffraction (XRD) spectroscopies were used to characterize DMBC700 and soil before and after adsorbing sulfate, respectively, and to analyze the mechanisms of adsorption. The results showed that the adsorption kinetics were well described by the pseudo-second-order model, whereas the Langmuir and Freundlich models fitted well with the equilibrium data. DMBC700 modification did not increase the adsorption capacity of light sierozem for sulfate. When the pH values of the initial solution were increased, all the adsorption capacities of sulfate onto DMBC700, light sierozem and light sierozem with DMBC700 decreased. The electrostatic interaction was the main force for the adsorption of sulfate onto DMBC700, whereas both electrostatic interaction and formation of poorly soluble CaSO_4_ were the main forces for adsorption of sulfate onto light sierozem. DMBC700 was found to have negative effect on sulfate adsorption onto light sierozem.

## Abstract 2

Amendment of dairy manure biochar exhibits negative effect on sulfate adsorption onto light sierozem.

## Graphical abstract

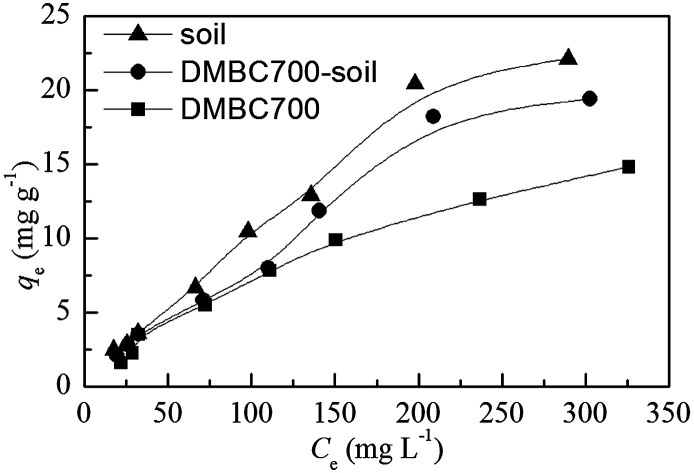

## Introduction

Sulfur is an essential nutrient; although its required amount is less than those of nitrogen, phosphorous and potassium, it plays an important role in plant growth. This is because some proteins and amino acids contain sulphur, and enzymatic reactions are based on sulfur sites; moreover, syntheses of chlorophyll, sitosterone, glutathione and coenzyme need a sulfur medium, and sulfur affects plant growth regulation, detoxification, defence and resistance as well as crop yield and quality.^1^

Biochar is a byproduct of the biomass pyrolysis process under limited oxygen conditions.^2,3^ Amendment of biochar into soils is considered to be a promising alternative for carbon sequestering, soil improvement and crop yield enhancement.^4^ In recent years, biochar has attracted increasing attention of scientists because of its adsorption ability for chemicals including nutrients. Thus, biochar amendment into soils might influence nutrient phytoavailability.^5–7^ Recently, the adsorption of nitrogen and phosphorus nutrients onto biochar has been investigated.^8–14^ Some positive results were observed with biochar enhancing NH_4_^+^, NO_3_^−^, and PO_4_^3−^ ion adsorption.^9,10,12^ However, a few limiting and even negative effects of biochars to fix NO_2_^−^, NO_3_^−^, and PO_4_^3−^ were reported.^13–18^ Comparatively, to the best of our knowledge, there are only few reports concerning the effects of biochars on sulfur adsorption and retention in soils.^19^ The situation of sulfur is not very clear because the adsorptive properties of biochar are largely dependent on the biomass feed type and pyrolysis conditions.

Thus, we prepared a biochar from dairy manure at 700 °C and selected light sierozem as the tested soil. Batch experiments were conducted to investigate sulfate adsorption onto biochar, soil, and biochar–soil mixtures. We aimed to determine the effects of dairy manure biochar on the adsorption of sulfate onto light sierozem and its mechanisms.

## Materials and methods

### Chemicals and materials

Sodium sulfate with analytical purity was obtained from Tianjin Guangfu Fine Chemical Institute, China. Deionized water was used in all experiments.

The dairy manure was collected from a farm in Anning District, Lanzhou City, China. The manure was air-dried, crushed by a grinder and passed through an 80 mesh sieve. The sample was put into a crucible, compacted, and covered with a lid. Then, the sample was heated in a muffle furnace at 700 °C for 6 h to pyrolyze the manure. After cooling to room temperature, the obtained biochar was passed through an 80 mesh sieve and labelled as DMBC700. The pH value of DMBC700 was measured using a pH meter (PHS-3C, Shanghai Electronic and Scientific Instrument Co., China) with 1 : 2.5 (w/w) suspension of the biochar in deionized water. The total C, H, O, N and ash contents in biochar were determined with an elemental analyzer (Vario EL, Elementar, Germany), and the atomic ratios were calculated. Brunauer–Emmett–Teller (BET) surface area was obtained from N_2_ adsorption at 77 K using Quantachrome Autosorb-1 (Quantachrome, USA). The basic physical and chemical properties of biochar are as follows: pH, 10.15; element composition (%): C 45.86, H 0.52, O 12.51, N 1.08, ash 39.28; atomic ratios: O/C 0.27, H/C 0.01, (O + N)/C 0.30; and specific surface area, 73.97 m^2^ g^−1^.

The light sierozem soil was sampled from the topsoil (0–20 cm) in a farmland in Yuzhong County, Lanzhou City, Gansu Province, China. After removing the impurities, the soil was air-dried, mixed thoroughly and passed through an 80 mesh sieve. The organic matter (OM) in the soil was analysed using the potassium dichromate oxidation method by a UV-1800 spectrophotometer (Shanghai Spectrum Instrument Co. Ltd., China). The pH value of the soil was measured on the pH meter with 1 : 5 (w/w) suspension of soil in deionized water. Cation exchange capacity (CEC) was determined according to the calcium acetate method (China NY/T1121.5-2006). The efficient sulfur (ES) was extracted using NaHCO_3_ and determined using the barium sulfate turbidity method. The basic physical and chemical properties of soil are as follows: organic matter (OM), 14.12%; pH, 7.97; cation exchange capacity (CEC), 5.65 cmol kg^−1^; and efficient sulfur (ES), 36.7 mg kg^−1^.

### Batch adsorption

The contact time, initial sulfate concentration and solution pH value were selected as the main factors, and batch adsorption experiments were conducted in the general process: a series of 0.1 g DMBC700, DMBC700-soil (1 : 9 (w/w)) or soil were weighed into 50 mL flasks containing 20 mL sodium sulfate solution. The samples were then put into a reciprocating shaker (THZ82, Jiangsu Jintan Youlian Instrument Institute, China) and equilibrated for a certain period at 25 °C. Then, the liquid–solid mixtures were filtered through a 0.45 μm membrane, and the sulfate concentration in filtrate was determined; here, the filtered single DMBC700 or light sierozem soil after adsorption in 400 mg L^−1^ of sodium sulfate solution for 16 h at 25 °C and pH 7 was air-dried and characterized by FTIR and XRD. The contact time, initial sulfate concentration and solution pH value were maintained as 20 h, 50 mg L^−1^ and 7 unless tested as a factor.

### FTIR and XRD characterization

FTIR spectra were obtained in the range of 500–4000 cm^−1^ of wave number on an IR spectrometer (Nicolet Nexus 870, USA). XRD was conducted on an X-ray diffractometer (PANalytical X'Pert Pro) over the 2*θ* range of 3–90° at a rate of 1°·min^−1^ with a step size of 0.02°.

### Analysis methods

The indirect atomic absorbance spectrometry (AAS) method^20^ was used to determine the sulfate concentration on an atomic absorbance spectrometer (SP-3520AAC2T1, Shanghai Spectrum Instrument Company, China). The adsorbed amount of sulfate (mg g^−1^) was calculated from the difference between the initial and equilibrium sulfate concentrations (mg L^−1^), solution volume (L) and adsorbent mass (g) in terms of the overall adsorbent mass. Nonlinear fitting was applied to obtain the regression parameters in kinetics and isotherm equations using OriginPro 8.0.

## Results and discussion

### Effect of contact time and adsorption kinetics

**Fig. 1:**
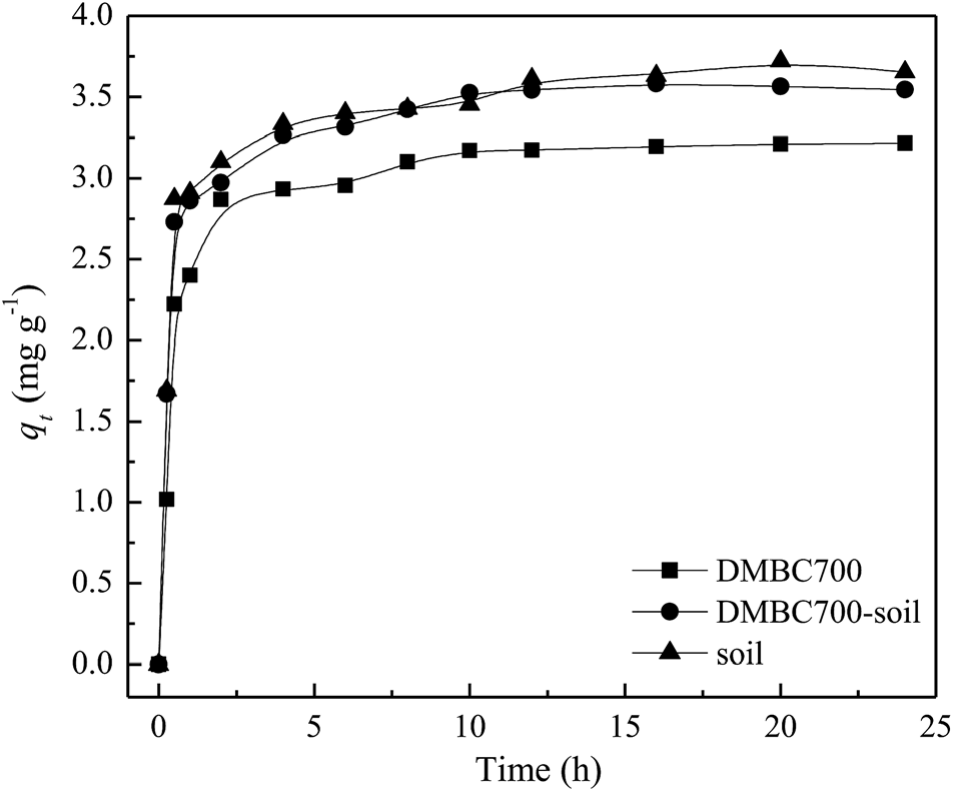
Effect of time on sulfate adsorption onto DMBC700, soil and soil with DMBC700 (*C*_0_ = 50 mg L^−1^, pH = 7, *T* = 25 °C).

The effects of contact time on absorption amounts of sulfate onto DMBC700, soil and soil with DMBC700 are shown in Fig. 1. It can be seen from Fig. 1 that the adsorption rate is fast in the initial 2 h, at which the adsorption amounts are 89.21, 84.84 and 83.89% of the equilibrium adsorption capacity. With increase in contact time, the adsorption amounts onto DMBC700, soil and soil with DMBC700 increased slightly and levelled off at about 3.65, 3.54 and 3.21 mg g^−1^, respectively. It was clear that the adsorption capacity of sulfate onto soil did not improve in the presence of DMBC700, indicating that DMBC700 may play a negative role in sulfate fixation after its considerable amendment into soils. Li *et al.* found that switchgrass and water oak biochars and biosolid biochar have limited and negative retention for NO_2_^−^ and NO_3_^−^.^13^ Liu *et al.* found that within a certain range of phosphate concentration in the equilibrium solution, the amount of phosphate adsorbed by three red soils decreased and the corresponding amount of phosphate desorbed increased with increasing amendment rate of rice straw biochar.^14^ The adsorption capacity of phosphate onto the engineered cow dung biochar (Mg-loaded) reached 345 mg g^−1^, which was significantly higher than those previously obtained under the same initial P concentrations.^12^ The adsorption capacities of biochars for anionic nutrients were largely different, which might be due to the biochar's surface components and properties.

Pseudo-first-order (1), pseudo-second-order (2), and Elovich (3) models were used to simulate the experimental kinetics, which can be expressed by the following equations:

1 d*q*_*t*_/d*t* = *k*_1_(*q*_e_ − *q*_*t*_)

2 d*q*_*t*_/d*t* = *k*_2_(*q*_e_ − *q*_*t*_)^2^

3 d*q*_*t*_/d*t* = *α* exp(−*βq*_*t*_)

Here, *q*_*t*_ (mg g^−1^) and *q*_e_ (mg g^−1^) are the adsorbed amounts of sulfate at time *t* (h) and at equilibrium, respectively; *k*_1_ (h^−1^) and *k*_2_ (g mg^−1^ h^−1^) are the adsorption rate constants of pseudo-first-order and pseudo-second-order models, respectively, *α* (mg g^−1^ h^−1^) is the initial sorption rate, and *β* (g mg^−1^) is the desorption constant. The fitting parameters of kinetic models are listed in Table 1. The *R*^2^ values (0.9426, 0.9343 and 0.9118) obtained from the pseudo-second-order model fitting for the adsorption of sulfate onto DMBC700, soil and soil with DMBC700 were higher than those of the pseudo-first-order and Elovich models. Moreover, the equilibrium adsorbed amounts of sulfate calculated from the pseudo-second-order model (*q*_e,cal_) were much closer to the experimental values shown in Fig. 1. This indicated that the pseudo-second-order model provides the best fit with the experimental data. Hafshejani *et al.* found a pseudo-second-order kinetic model, which could fit well with the experimental data for the adsorption of nitrate onto modified sugarcane bagasse biochar.^8^ Similar results were also reported by Takaya *et al.* for the adsorption of phosphate ions onto chars.^10^

**Table 1:** Kinetic parameters for sulfate adsorption onto DMBC700, soil and soil with DMBC700

Model	Parameter	DMBC700	DMBC700-soil	Soil
Pseudo-first-order	*q*_e,cal_ (mg g^−1^)	3.11	3.40	3.46
	*k*_1_ (h^−1^)	2.10	2.94	3.07
	*R* ^2^	0.919	0.837	0.828
Pseudo-second-order	*q*_e,cal_ (mg g^−1^)	3.26	3.55	3.61
	*k*_2_ (g mg^−1^ h^−1^)	0.864	1.21	1.24
	*R* ^2^	0.943	0.934	0.912
Elovich	*α* (mg g^−1^ h^−1^)	0.022	0.001	0.003
	*β* (g mg^−1^)	0.003	0.004	0.003
	*R* ^2^	0.801	0.841	0.819

### Effect of sulfate concentration and adsorption isotherms

**Fig. 2:**
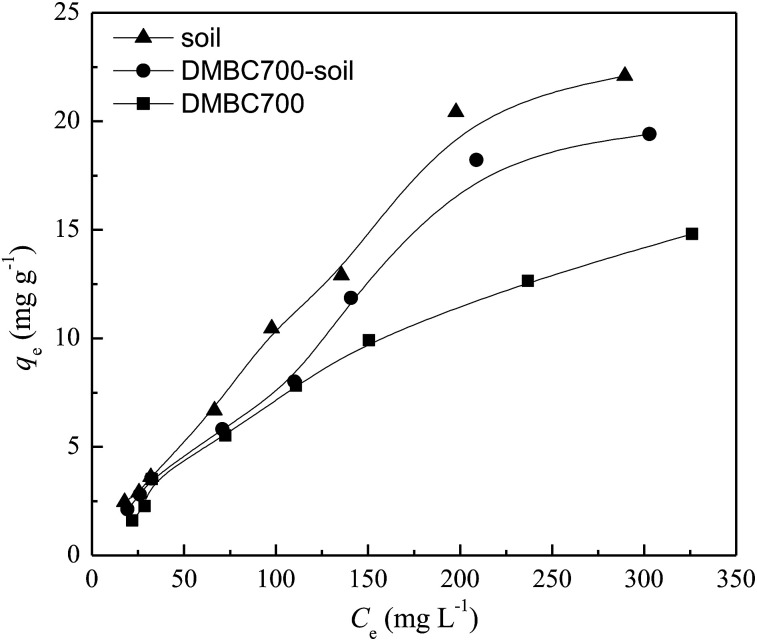
Effect of equilibrium concentration on sulfate adsorption onto DMBC700, soil and soil with DMBC700 (*t* = 16 h, pH = 7, *T* = 25 °C).

The relationships between the adsorption capacities of DMBC700, soil and soil with DMBC700 for sulfate and the equilibrium sulfate concentrations are shown in Fig. 2. The adsorption amounts of sulfate onto DMBC700, soil and soil with DMBC700 increased with the sulfate concentration. This might be due to the higher concentration gradients in the systems, which resulted in higher occupation of the reactive sorption sites.^10,21^ Clearly, the adsorption amounts of sulfate onto soil were larger than those onto DMBC700 and soil with DMBC700.

Three models Langmuir (4), Freundlich (5), and Temkin (6)^22^ were used to fit the sulfate adsorption equilibrium described in Fig. 2:

4 *q*_e_ = *q*_m_*K*_L_*C*_e_/(1 + *K*_L_*C*_e_)

5 *q*_e_ = *K*_F_*C*_e_^1/*n*^

6 *q*_e_ = *B* ln *AC*_e_

Here, *q*_e_ is the equilibrium adsorption capacity of sulfate (mg g^−1^), *C*_e_ is the equilibrium sulfate concentration in an aqueous solution (mg L^−1^), *K*_L_ is the Langmuir constant (L mg^−1^), *K*_F_ (mg g^−1^ L mg)^1/*n*^ is the Freundlich constant, *n* (dimensionless) is the adsorption intensity factor, *q*_m_ is the maximum adsorption capacity (mg g^−1^), *B* is the equilibrium binding constant (L mol^−1^), and *A* is a constant related to heat adsorption. The regression results are listed in Table 2. According to the obtained *R*^2^ values, sulfate adsorption onto DMBC700, soil and soil with DMBC700 could fit well with the Freundlich and Langmuir models. In some cases, it was found that the Langmuir model was better than the Freundlich one for fitting phosphate,^9,11,21^ nitrate^8^ and ammonium^13^ species on biochars, while the Freundlich model was more suitable for fitting the adsorption of phosphate on several chars.^10^ Liu *et al.* found that depending on the soil pH, the Temkin isotherms for P sorption in low-pH soils revealed high *R*^2^ values.^14^

**Table 2:** Isotherm parameters for sulfate adsorption onto DMBC700, soil and soil with DMBC700

Model	Parameter	DMBC700	DMBC700-soil	Soil
Langmuir	*q*_e,cal_ (mg g^−1^)	78.1	78.4	83.0
	*K*_L_ (L mg^−1^)	0.00087	0.00088	0.00096
	*R* ^2^	0.960	0.978	0.986
Freundlich	*K*_F_ (L mg^−1^)	0.219	0.121	0.144
	*N*	1.41	1.17	1.18
	*R* ^2^	0.942	0.975	0.981
Temkin	*A* (L mg^−1^)	0.039	0.034	0.033
	*B*	5.01	6.78	7.77
	*R* ^2^	0.971	0.923	0.913

### Effect of solution pH value and adsorption mechanisms

**Fig. 3:**
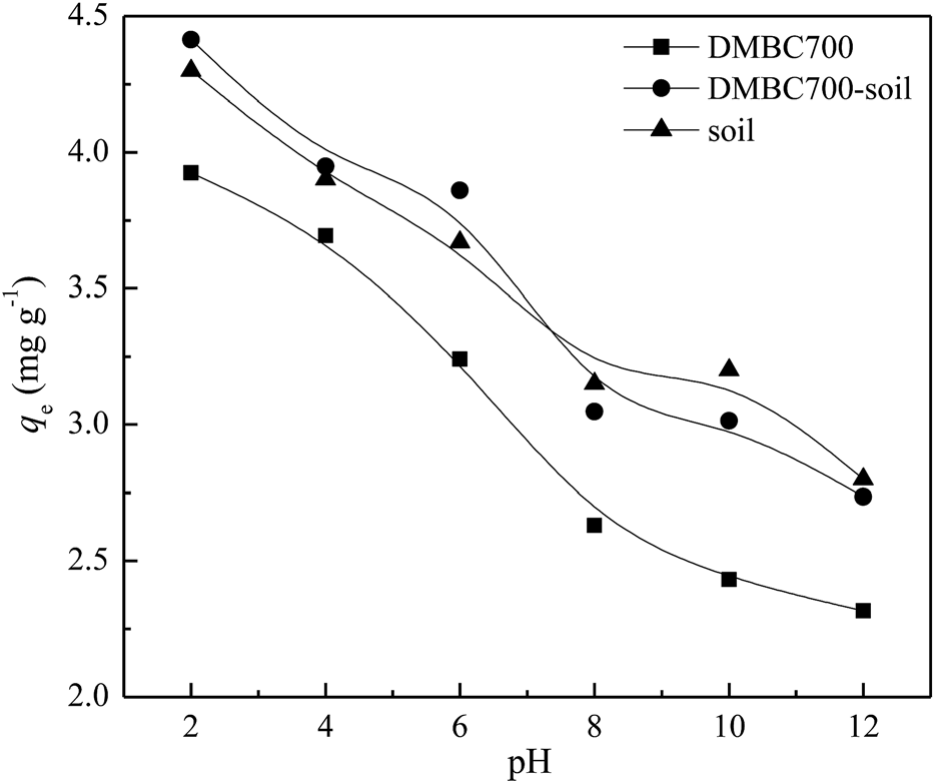
Effect of pH value on sulfate adsorption onto DMBC700, soil and soil with DMBC700 (*C*_0_ = 50 mg L^−1^, *t* = 16 h, *T* = 25 °C).

Fig. 3 shows that the sulfate adsorption amounts change with the initial solution pH values. The adsorbed amounts of sulfate onto DMBC700, soil and soil with DMBC700 decreased gradually when the pH values increased from 2 to 12. This might be attributed to the electrostatic interactions between sulfate and the charged sorbent surfaces. In general, the charges loaded on the soil particle surfaces are classified as variable and constant ones. The later derived from the isomorphous replacement in mineral formation is not affected by the change in pH. However, the former arising from organic matters, metal oxides, hydrated metal oxides, *etc.* changes with the solution pH value, due to which the total charge of soil particle surfaces can be positive, zero or negative.^23^ Various functional groups on the surfaces of biochar may influence sorption by the nature of their surface charge. Similar to that observed for oxide surfaces, the charge on the functional groups may change depending upon the pH value of the solution, thus affecting sorption behaviour.^4,24^ At lower pH values, more positively charged sites are present on DMBC700 and soil surfaces. Thus, more sulfate ions can be adsorbed.^25,26^ At pH 2, the adsorbed amounts of sulfate on DMBC700, soil and soil with DMBC700 were 3.93, 4.30 and 4.41 mg g^−1^, respectively. With increase in pH, the soil and DMBC700 surfaces became more negatively charged and thus, the adsorbed amounts of sulfate decreased. Meanwhile, the reduction in adsorbed amounts of sulfate was partially attributed to the competition of hydroxyl ion adsorption onto soil and DMBC700 with the increase in pH. At pH 12, the adsorbed amounts of sulfate on DMBC700, soil and soil with DMBC700 were 2.31, 2.80 and 2.73 mg g^−1^, respectively. A similar trend was also found in recent reports for nitrate adsorption onto sugarcane bagasse biochar^8^ and phosphate adsorption^11^ onto chars.

**Fig. 4:**
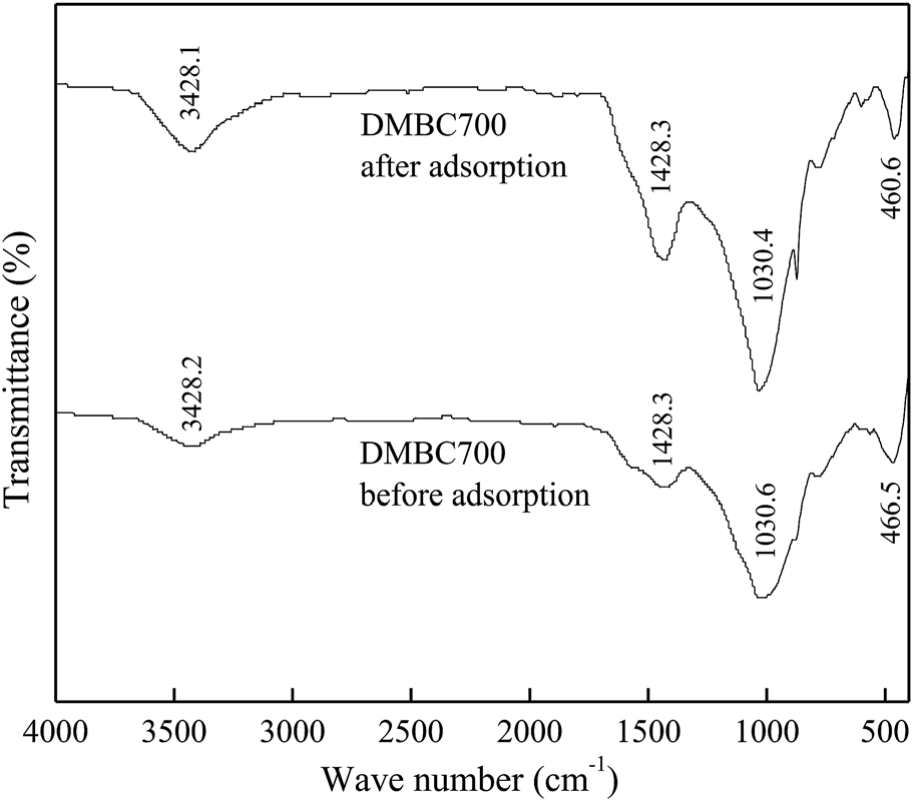
FTIR spectra of DMBC700 before and after adsorption.

**Fig. 5:**
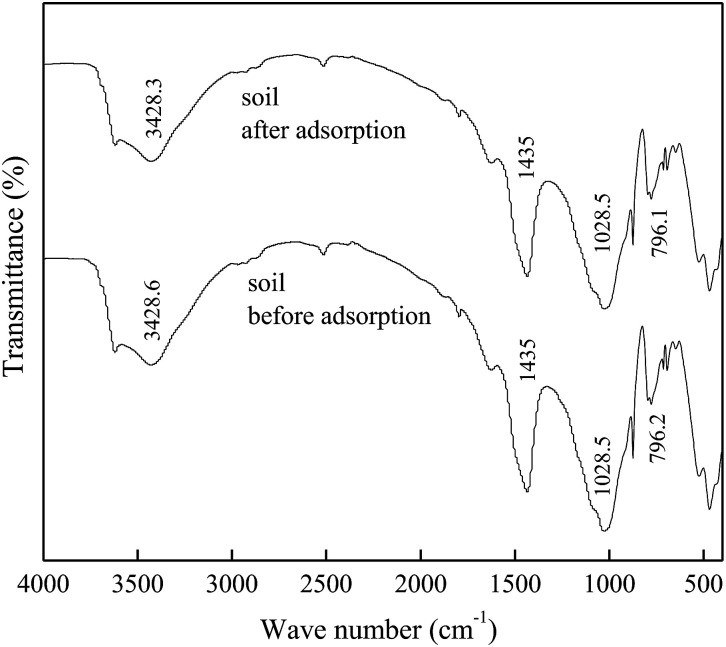
FTIR spectra of soil before and after adsorption.

The FTIR spectra of DMBC700 and soil before and after adsorption of sulfate are shown in Fig. 4 and 5, respectively. It is found that the main transmittance peak intensity and the corresponding wave number did not change significantly. It was indicated that the sulfate adsorption was not due to the interaction between the surface functional groups and sulfate ions.

**Fig. 6:**
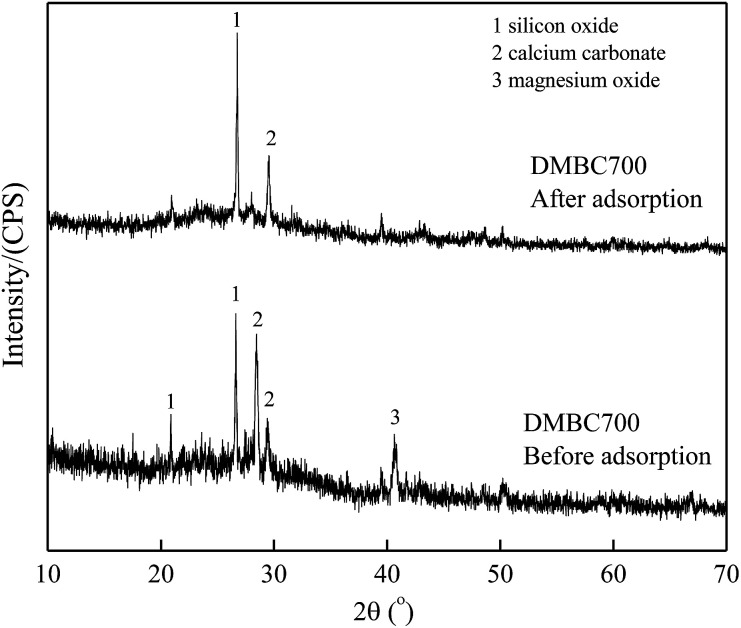
XRD spectra of DMBC700 before and after adsorption.

**Fig. 7:**
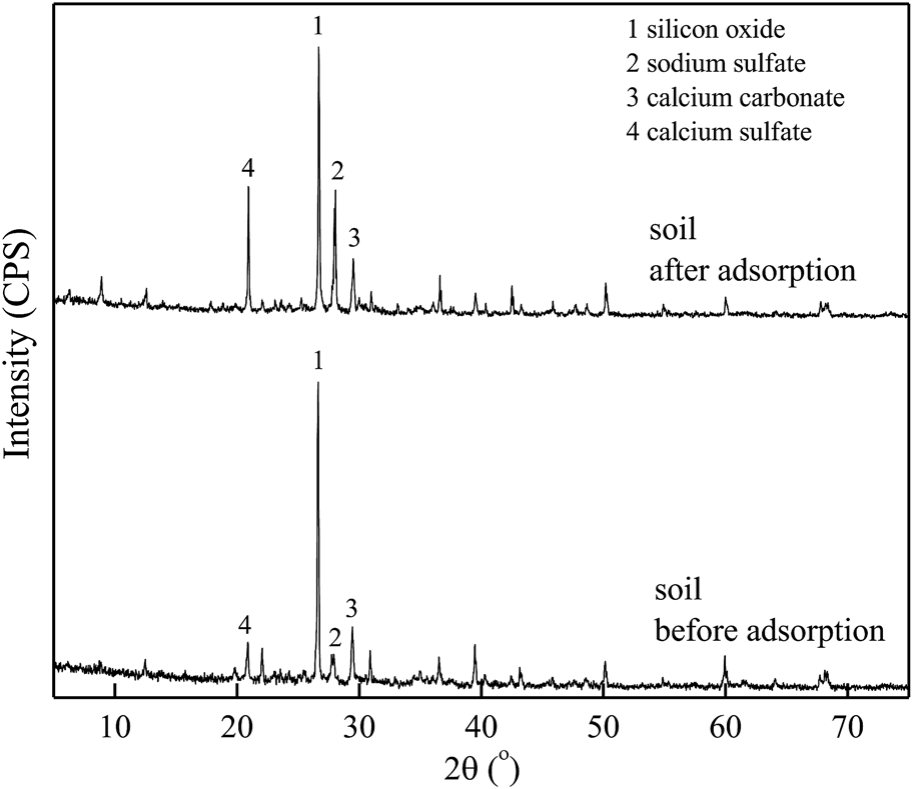
XRD spectra of soil before and after adsorption.

Fig. 6 and 7 show the XRD spectra of DMBC700 and soil before and after adsorbing sulfate, respectively. DMBC700 showed a somewhat crystalline structure with a higher mineral content. The presence of quartz, calcite and periclase was confirmed. However, no new mineral peak occurred in the XRD spectrum of DMBC700 after adsorbing sulfate, indicating that DMBC700 and sulfate ions did not react with the newly formed precipitates. The main mineral components of soil were quartz, calcite, kaolinite, bog iron ore and muscovite. A strong and broad peak at 2*θ* = 28.07° indicated the presence of sodium sulfate, which might be due to the reaction between the irregular clay mineral surface and sulfate ions. After sulfate adsorption, a strong CaSO_4_ peak occurred at 2*θ* = 20.54°. Therefore, it was concluded that one of the sulfate ion adsorptions could be attributed to the formation of poorly soluble calcium sulfate. As a calcareous soil, the carbonate content in light sierozem is high. Sulfate ions could thus react with the calcium ions in calcium carbonate to form a precipitate.^23^

## Conclusions

The kinetics of sulfate adsorption onto DMBC700, soil and soil with DMBC700 could be described using a pseudo-second-order model, while the isotherms could fit well with Langmuir and Freundlich models. On the basis of the results of the effect of pH values on adsorption and FTIR and XRD analyses before and after adsorption, it was shown that the main driving force of sulfate adsorption onto DMBC700 was the electrostatic interaction; moreover, for sulfate adsorption onto light sierozem, both electrostatic interaction and formation of poorly soluble CaSO_4_ were the major forces. However, DMBC700 amendment did not enhance the adsorption capacity of light sierozem for sulfate ions.

## Conflicts of interest

There are no conflicts to declare.

## Supplementary Material
